# Trop-2 Therapy in Metastatic Triple-Negative Breast Cancer in Italy: Clinical Opportunity and Regulatory Pitfalls

**DOI:** 10.3390/jpm11111211

**Published:** 2021-11-16

**Authors:** Sara Bravaccini, Roberta Maltoni

**Affiliations:** IRCCS Istituto Romagnolo per lo Studio dei Tumori (IRST) “Dino Amadori”, Via Maroncelli 40, 47014 Meldola, Italy; roberta.maltoni@irst.emr.it

**Keywords:** sacituzumab govitecan, metastatic triple-negative breast cancer, Trop-2, guidelines, patient selection

## Abstract

Trop-2 is an ideal candidate for targeted therapeutics because it is a transmembrane protein with an extracellular domain overexpressed in a wide variety of tumors, and is upregulated in normal cells. Consequently, several Trop-2-targeted drugs have recently been developed for clinical use, such as anti-Trop-2 antibodies. Sacituzumab govitecan, a Trop-2-directed antibody and topoisomerase inhibitor drug conjugate, was recently approved by the Food and Drug Administration (FDA) and European Medicines Agency (EMA) for the treatment of metastatic triple-negative breast cancer and metastatic urothelial cancer. In Italy, this treatment cannot be used in clinical practice because it has not yet been approved by the Agenzia Italiana del Farmaco (AIFA, Rome, Italy). In Italy, this is not a new problem, in fact, when a new compound is approved by the U.S. and Europe, there is often a delay in its approval for use. The adoption of universal guidelines and the standardization of Trop-2 evaluation is urgently needed.

## 1. Introduction

The World Health Organization (WHO) estimates for Italy an incidence of 55.133 new breast cancer (BC) cases in 2020, 15–20% of them are triple-negative breast cancer (TNBC), while in the U.S., 276.480 are new BC case estimates [1,2]. Patients with metastatic triple-negative breast cancer (mTNBC) (defined by a lack of tumor-cell expression of the estrogen receptor, progesterone receptor, and human epidermal growth factor receptor 2 [HER2]) have unfavorable outcomes [3,4]. Despite immunotherapy having demonstrated promising first-line clinical activity, single-agent chemotherapy represents the standard for previously treated (beyond first-line) mTNBC, even if it is associated with low response rates and short progression-free survival [5,6]. Trop-2 is a transmembrane calcium signal transducer that is highly expressed in multiple tumor types, including TNBC. The possibility to treat Trop-2 positive mTNBC patients is fascinating and opens up new possibilities of treatment in this prognostic unfavorable population.

### 1.1. Sacituzumab Govitecan

Sacituzumab govitecan is an antibody drug conjugate (ADC) composed by a humanized RS7 antibody targeting Trop-2 linked with SN38, a topoisomerase I inhibitor, the active metabolite of irinotecan with 100–1000-fold higher potency with respect to the parent drug [7,8]. SN-38 interacting with topoisomerase I prevents relegation of topoisomerase I–induced single strand breaks and leads to DNA damage, apoptosis, and cell death [7,8]. Sacituzumab govitecan gives both direct cytotoxicity and antibody-dependent cellular cytotoxicity [9,10]. The pharmacokinetics of sacituzumab govitecan was studied in patients with several pretreated metastatic epithelial cancers in a phase I/II study [10]. Sacituzumab govitecan was administered at starting doses of either 8 or 10 mg/kg intravenously on days 1 and 8 of 21-day cycles in the phase II component of the study, and it was observed that peak antibody concentrations increased proportionally with continued treatment in the group of 10 mg/kg. Within 3 days of treatment, levels of SN38 were largely cleared at a rate of about 50% per day [4]. Of the 84% of patients who received sacituzumab govitecan, and had *UGT1A1* genotype results, the incidence of grade 4 neutropenia was 26% in patients homozygous for the UGT1A1*28 allele, 13% in patients heterozygous for the UGT1A1*28 allele, and 11% in patients homozygous for the wild type allele [9]. Despite it is not recommended routine *UGT1A1* genotyping prior to starting sacituzumab, patients who are known to have reduced *UGT1A1* activity should be monitored closely for severe neutropenia [9].

### 1.2. Trop-2

Trop-2 (also called epithelial glycoprotein-1, gastrointestinal antigen 733-1, membrane component surface marker-1, and tumor-associated calcium signal transducer-2) is a product of the *TACSTD2* gene located at 1p32.1 [11]. Trop-2 is a 40-kDa glycoprotein that was described as transducer of intracellular calcium signaling [12,13]. It contains a 274-amino-acid extracellular epidermal growth factor-like repeat portion with three domains, a cysteine-rich domain, a thyroglobulin type-1 domain, and a cysteine-poor domain [11].

The Trop-2 protein interacts with multiple cellular regulators, including Insulin-like growth factor 1, claudin-1, claudin-7, cyclin D1, and protein kinase C [11]. In addition, various transcription factors closely interact with the *TACSTD2* gene, such as *HNF4A*, *TP63/TP53, WT1, ERG, HNF1A/TCF-1*, and *FOXP3* [14,15].

Trop-2 expression was first described in trophoblasts (placenta) and fetal tissues (e.g., lung) and subsequently described in the normal stratified squamous epithelium of the skin, esophagus, uterine cervix, and tonsil crypts [16] even if many normal tissues lack or show low Trop-2 protein expression (for example colon, kidney, liver, lung, prostate, and breast) [11].

Aberrant Trop-2 overexpression has been described in solid cancers, including those with low Trop-2 expression in their normal counterparts (e.g., colorectal, renal, lung, and breast carcinomas) [11,17]). Trop-2 plays a role in tumor progression, given its active interplay with several key molecular pathways traditionally associated with cancer development and progression [11]. High Trop-2 expression usually gives a poor outcome [18]. In a meta-analysis by Zeng et al. that included 2569 cancer patients (reflecting 13 common solid malignancies), increased Trop-2 expression was particularly associated with poor overall survival (OS) and disease-free survival (DFS) outcomes in patients with gastrointestinal and gynecological malignancies [18]. Despite the several limitations of this study, such as inconsistency in Trop-2 detection and criteria of Trop-2 positivity, the authors stated that a frequent Trop-2 expression in the majority of solid tumors and its association with a poor prognosis provided a good rationale to target Trop-2 for therapeutic purposes [18]. Trop-2 expression has also been described in some rare and aggressive malignancies, such as salivary duct carcinomas [19], anaplastic thyroid carcinomas [20], uterine/ovarian carcinosarcomas [21,22], and neuroendocrine carcinoma (NEC) of the prostate [23]. In prostate (NEC), Trop-2 seems to closely interplay with the Poly ADP-ribose polymerase enzyme promoting neuroendocrine phenotype and aggressiveness of prostate cancer [24].

### 1.3. Preclinical and Clinical Studies

Sacituzumab govitecan has been shown to significantly inhibit tumor growth in translational models of BRCA-mutant human TNBC, and is hypothesized to confer synthetic lethality to TNBC [25]. This drug is a conjugate of the humanized anti-Trop-2 monoclonal antibody linked to SN-38, the active metabolite of irinotecan [26]. SN-38 is too toxic to be administered directly to patients, but linkage to an antibody allows the drug to specifically target cells containing Trop-2. Research continues into the differential expression of Trop-2 in cancer and normal epithelial cells [27]. Zhao et al. reported a correlation between Trop-2 mRNA and protein expression levels and several clinical prognostic indicators in breast cancer, including lymph node status, metastasis, stage, and estrogen receptor/progesterone receptor/HER2 expression. The authors demonstrated that Trop-2 is a potential biomarker of the epithelial-mesenchymal transition process in breast cancer [28]. Gu et al. reported that Trop-2 knockdown decreased cell migration and proliferation, suggestive of a therapeutic benefit of Trop-2 [29]. Trop-2 is expressed in all breast cancer subtypes and linked to poor prognosis including decreased survival. High Trop-2 membrane expression has been observed in TNBC. In particular, Bardia et al. reported that, of 48 translational models of primary and mTNBC, around 88% showed moderate to strong Trop-2 staining, the majority also expressing Trop-2 in >50% of tumor cells [30].

Sacituzumab govitecan has shown a potential for greater efficacy in tumors with relatively high Trop-2 expression, and increased antitumor activity of sacituzumab govitecan has been seen in tumor xenografts derived from Trop-2-overexpressing breast cancer clones.

A confirmatory multicenter, randomized, phase III trial Ascent study (NCT02574455) recruited patients in North America and Europe to compare sacituzumab govitecan with the physician’s choice of four single-agent types of chemotherapy (capecitabine, gemcitabine, vinorelbine, and eribulin) in patients with mTNBC that is refractory or relapsed after at least two previous forms of chemotherapy (including a taxane) [31].

It has been demonstrated on patients with pretreated mTNBC, a significant superiority of sacituzumab govitecan over chemotherapy in terms of survival and a tolerable safety profile, with a median progression-free survival of 5.6 vs. 1.7 months, and an OS of 12.1 vs. 6.7 months, respectively [31]. Sacituzumab govitecan had efficacy with a 33% response rate in a heavily pretreated population of patients with mTNBC. Diarrhea and myelosuppression were the primary adverse events, and discontinuation rates were low [31].

At the 2020 San Antonio Breast Cancer Conference, in a presented biomarker analysis, it was shown that the benefit in favor of sacituzumab govitecan was maintained independently of the degree of Trop-2 expression, even if the smallest progression free survival (PFS) and overall survival (OS) difference was observed in patients with low levels of Trop-2 expression in the tumor (PFS: Trop-2 high 6.9 vs. 2.5 months, Trop-2 medium 5.6 vs. 2.2 months, Trop-2 low 2.7 vs. 1.6 months; OS: Trop-2 high 14.2 vs. 6.9 months, Trop-2 medium 14.9 vs. 6.9 months, Trop-2 low 9.3 vs. 7.6 months) [32]. Superiority of sacituzumab govitecan was maintained independently from germline BRCA1/2 mutation status. These findings suggest that sacituzumab govitecan is an important option in the treatment of TNBC. However, in the subgroup of patients with stable brain metastases (BM) at baseline (*n* = 61), sacituzumab govitecan was not superior to conventional chemotherapy in terms of PFS and OS [33]. A conclusion on the activity of sacituzumab govitecan in BM cannot be drawn as patients with active brain metastases (i.e., newly diagnosed or progressing after prior radiotherapy) were excluded from the study. A subgroup analysis of biomarker expression performed by Bardia et al. showed that the superior clinical benefit of sacituzumab govitecan over single-agent chemotherapy of the physician’s choice (TPC) in previously treated mTNBC was independent of the level of Trop-2 expression [30]. However, a better outcome was observed in patients undergoing sacituzumab govitecan who had a medium/high Trop-2 H score (vs. low Trop-2 H score) compared to those treated with TPC. However, the number of patients analyzed was too low to be able to draw any definitive conclusions [30]. Moreover, problems encountered in Trop-2 detection may be due to the methodology used in terms of antibody type and H scoring system. Its evaluation by liquid biopsy could also be useful to monitor disease and response to therapy.

Multiple studies of sacituzumab govitecan involving patients with breast cancer are ongoing, including evaluation of the agent as neoadjuvant therapy in early TNBC (NeoSTAR (ClinicalTrials.gov number, NCT04230109), as adjuvant therapy (GBG102-SASCIA (EudraCT number, 2019-004100-35), in the metastatic subset in combination with immunotherapy regimens (Morpheus-TNBREAST CANCER (ClinicalTrials.gov number, NCT03424005) and Saci-IO TNBC (NCT04468061)) or with a PARP inhibitor (NCT04039230) in advanced TNBC, and in hormone receptor–positive and HER2-negative metastatic breast cancer (TROPiCS-02) [31]. Sacituzumab govitecan is also being studied in the treatment of ER-positive metastatic breast cancer. Trop-2 expression has also been described in estrogen receptor (ER)-positive breast cancers [34], although it appeared to be lower than in TNBC. This was observed in breast cancer cell lines and in breast tumor samples.

Recent data also indicate a promising therapeutic activity of sacituzumab govitecan in patients with luminal (ER+) subtype of breast cancer. Thus, a phase I/II single-arm basket trial involving 54 heavily pretreated patients with ER+/HER2- breast cancer revealed convincing therapeutic effects of sacituzumab govitecan [34]. At a median follow-up of 11.5 months, the overall response rate (ORR) was 31.5%, median duration of response (DOR) was 8.7 months, and median PFS was 5.5 months, while the median OS was 12 months. A new phase III clinical trial (TROPiCS-02 study, NCT03901339), evaluating sacituzumab govitecan versus standard treatment in ER+/HER2- metastatic breast cancers, has also been initiated recently and is expected to provide data in the near future [34].

### 1.4. Regulatory Issue

Sacituzumab govitecan was recently approved from the Food and Drug Administration (FDA, Silver Spring, MD, USA) and European Medicines Agency (EMA, Amsterdam, The Netherlands) for the treatment of mTNBC and metastatic urothelial cancer [26]. In particular, for breast cancer patients, it is approved in those with unresectable locally advanced or mTNBC who have been treated with two or more prior systemic therapies, at least one of them for metastatic disease [35]. However, although the results of a phase I/II trial (NCT 01631552) led to the accelerated approval of intravenous sacituzumab govitecan in the U.S. in April 2020 for the treatment of patients with mTNBC after >2 prior therapies for metastatic disease, 13 years have passed since the first preclinical trial (October 2007) on this drug was carried out [26]. Furthermore, continued approval will require evidence of a clinical benefit in a confirmatory phase III trial.

Once the effect of the drug on the identified target has been ascertained in the laboratory and an acceptable degree of safety has been defined for use, it is necessary to verify its real tolerability and efficacy on humans. The whole clinical experimentation must be conducted according to the Good Clinical Practice (GCP), and this process is verified by AIFA Italy. The path of the clinical trial is based on four phases (I, II, III, and IV), which have become “standard” in each country.

Phase I clinical trials answered the fundamental question: is the drug safe?

They are carried out in a few selected centers on a small number of healthy volunteers. The main purpose of these studies is to provide a preliminary assessment on the safety and distribution of the drug in the body and to confirm the data obtained in humans, in the preclinical research phase, in the laboratory and on animals. In the case of serious pathologies (for example oncological pathologies), phase I studies can be conducted directly on patients.

Phase II clinical trials answer the fundamental question: does the drug work?

They are also referred to as “pilot therapeutic studies”. Their purpose is in fact to capacity the activity of a potential drug in patients affected by a disease and to confirm its safety. These studies are on a limited number of people, usually a few hundred, and often compare (comparative studies) the effectiveness of the drug being evaluated with another medicine or with a non-effective substance (placebo). In this phase it is decided which dose is the most effective and best tolerated by the patient.

Phase III clinical trials answer the fundamental questions: how effective is the drug? Does it have any more benefits than similar drugs already on the market?

They are defined as “therapeutic-confirmatory” studies performed on larger groups of patients in order to determine the relationship between the safety and efficacy of the new drug even for prolonged treatment over time. The characteristics of the most frequent adverse reactions and undesirable effects that are detected by comparing the drug with a placebo or with other drugs already in use are then investigated. Thousands of patients in different countries are typically involved in this phase.

On the basis of the studies conducted in the first three phases, the competent health authorities verify the safety and efficacy of the new drug and authorize its registration and marketing (AIC) (phase IV clinical trials), establishing its price on the market [36].

**Figure 1:**
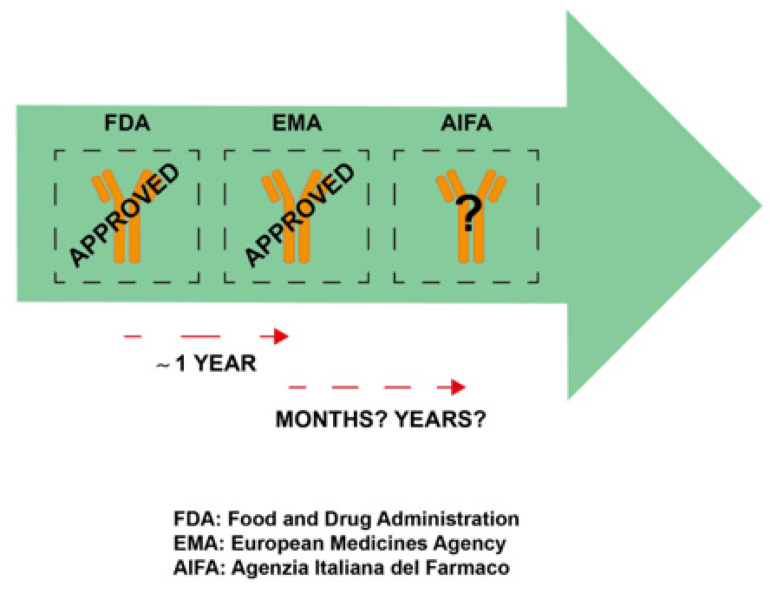
Graphical abstract on time of approval for sacituzumab govitecan by regulatory agencies.

In Italy, the treatment with sacituzumab govitecan cannot be used in clinical practice due to the lack of AIFA approval (Figure 1). This is a recurring issue each time a new compound is approved by the FDA in the U.S. and by the EMA in Europe, given that there is a delay in AIFA approval. After FDA approval, in Europe, access of a new drug to the patient is the final moment in a long and complex “journey” that starts with the EMA, involves the AIFA, and finally reaches the individual regions [35]. In this process, the EMA has to evaluate the drug from the scientific point of view, AIFA makes a decision in terms of reimbursement, while each individual regions carry out, in a non-homogeneous way and with different procedures, further evaluation before actually making the drug available to Italian patients.

The authorization procedure begins with an application for registration of a drug to the EMA, which evaluates the product and formula, by the Committee for Medicinal Products for Human Use (CHMP) and it gives a favorable or unfavorable opinion. This opinion is then ratified in the European Commission Decision, authorizing the placing of the drug on the market. At this point, the drug is registered in all countries of the European Union, and it is theoretically available for patients. The national phase now begins, in which AIFA must decide if and under what conditions (price and type of patients) the drug could be reimbursed, and then made available to the patient free of charge. During this period, the companies can render the drug available in any case, but the free of charge use of the drug is decided by the single hospital, Azienda Sanitaria Locale (AUSL), or region.

The decisions within the AIFA are taken by the Technical-Scientific Commission (CTS) and by the Prices and Reimbursement Committee (CPR). Based on the decisions expressed by the two Commissions, AIFA gives an authorization, which is then published in the Gazzetta Ufficiale della Repubblica Italiana (GURI). Afterward, the drug should be available for Italian citizens, but the journey to the patient is not finished, because the individual Italian regions often slow down the actual availability of drugs for patients via additional bureaucratic procedures.

This path goes on up to 3 years. Fortunately, Italy, like the other major European nations, has allowed—for various procedures—to make a drug free of charge, available before it is officially authorized, in the event that this can bring important benefits to patients with severe diseases [35].

In particular, for sacituzumab govitecan use in clinical practice, a long time (13 years) has passed since the first preclinical study to the FDA approval and even more time is needed for its approval in Italy. Unfortunately, given that this drug is approved in the subset of metastatic breast cancer patients, none of the patients can wait all this time. This is an issue that needs to be solved with urgency, to provide these patients with the possibility of treatment during their lives. The patient with metastatic cancer has to be considered a healthcare emergency.

This emergency involves all new drugs and all oncological disease (hematologic malignancies and solid tumors) in both neoadjuvant, adjuvant, and metastatic settings.

In Italy, the WHO estimates an incidence of 55.133 new breast cancer cases (females) in 2020 [2]. Given that about 15–20% of them are TNBC, of whom 88% express moderate-strong Trop-2 expression in primary or metastatic tumor, this means that a large number of patients will not receive any antibody-drug conjugate treatments.

## 2. Discussion

Larger studies are needed to establish whether Trop-2 expression is correlated with a specific TNBC patient subgroup on the basis of molecular stratification (i.e., basal-like 1, basal-like 2, mesenchymal, mesenchymal stem-like, immunomodulatory, and luminal androgen receptor), and to determine its real value as a predictive biomarker, considering other methods and criteria of classification for its use in the clinical practice. The adoption of universal criteria, guidelines to recruit patients, and the standardization of biomarker detection methods are mandatory issues.

The adoption of universal guidelines is warranted and the standardization of Trop-2 evaluation is urgently needed.

Other questions concern the potential efficacy of sacituzumab govitecan in earlier treatment lines and its combination with immune checkpoint inhibitors or PARP inhibitors. Clinical trials are ongoing in different tumor subtypes and in different disease phases [31,37]. Further research is warranted to see whether there are other biomarkers even more predictive of sacituzumab govitecan efficacy than Trop-2. The delay in sacituzumab govitecan approval in Italy and the biological difficulties in Trop-2 detection have prevented the use of this drug in potentially responsive patients.

## 3. Conclusions

The process of drug development, from the preclinical phase to drug availability, requires a mean of 10–12 years. The acceleration of the entire process is possible, as evidenced by the recent COVID-19 heath emergency—the rapid commercialization of vaccines is the only true “weapon” that will permit us to win the battle against the pandemic. We suggest considering the possibility of rendering this process faster, for every drug, including drugs used in oncology, so a larger number of patients could benefit from innovative, safe, and effective treatments.

## Data Availability

The authors confirm that no new data were created in this study.

## Abbreviations

FDA	Food and Drug Administration
EMA	European Medicines Agency
AIFA	Agenzia Italiana del Farmaco
